# A Glycomarker for Short-term Prediction of Hepatocellular Carcinoma: A Longitudinal Study With Serial Measurements

**DOI:** 10.1038/s41424-018-0050-3

**Published:** 2018-09-20

**Authors:** Yu-Ju Lin, Chia-Ling Chang, Liang-Chun Chen, Hui-Han Hu, Jessica Liu, Massaki Korenaga, Yu-Han Huang, Chin-Lan Jen, Chien-Yu Su, Nao Nishida, Masaya Sugiyama, Sheng-Nan Lu, Li-Yu Wang, Yong Yuan, Gilbert L’Italien, Hwai-I Yang, Masashi Mizokami, Chien-Jen Chen, Mei-Hsuan Lee

**Affiliations:** 1 Institute of Clinical Medicine, National Yang-Ming University, Taipei, Taiwan; 2 Genomics Research Center, Academia Sinica, Taipei, Taiwan; 3 Faculty of Medicine, National Yang-Ming University, Taipei, Taiwan; 4 California Perinatal Quality Care Collaborative, Palo Alto, California USA; 5 Perinatal Epidemiology and Outcomes Research, Division of Neonatology, Department of Pediatrics, School of Medicine, Stanford University and Lucile Packard Children’s Hospital, Palo Alto, California USA; 6 Kohnodai Area Research Center for Hepatitis and Immunology, National Center for Global Health and Medicine, Ichikawa, Japan; 7 Genome Medical Sciences Project, National Center for Global Health and Medicine, Ichikawa, Japan; 8 Department of Gastroenterology, Chang-Gung Memorial Hospital, Kaohsiung, Taiwan; 9 Department of Medicine, Mackay Medical College, Taipei, Taiwan; 10 Global Health Economics and Outcome Research, Bristol-Myers Squibb, Princeton, NJ USA; 11 Biohaven Pharmaceuticals, New Haven, CT USA; 12 Academia Sinica, Taipei, Taiwan

## Abstract

**Objectives:**

*Wisteria floribunda* agglutinin-positive human Mac-2-binding protein (WFA^+^-M2BP) is a glycomarker. The present community-based long-term follow-up study repeatedly determined the serum WFA^+^-M2BP level and examined its short- and long-term associations with hepatitis C virus (HCV)-related hepatocellular carcinoma (HCC).

**Methods:**

A total of 921 participants with antibodies against HCV seropositive, but seronegative for hepatitis B surface antigen were enrolled from seven townships in Taiwan during 1991–1992. The participants were regularly followed and their serum WFA^+^-M2BP levels were measured at baseline and follow-up. HCC was ascertained through active follow-up and computerized data linkage with the National Cancer Registration System until December 31, 2013. Cox proportional hazards and logistic regression models were applied to estimate the magnitude of associations between serum WFA^+^-M2BP levels and HCC.

**Results:**

During a median follow-up of 21.7 years, 122 new-onset HCC cases were identified. Elevated serum WFA^+^-M2BP levels were associated with an increased risk of HCC (*p* < 0.001). Patients with increasing changes in serum WFA^+^-M2BP levels, relative to their baseline levels, had a 4.36-fold risk of HCC. The areas under receiver operating curves (AUROCs) of WFA^+^-M2BP for predicting HCC showed that the prediction efficacy was significantly higher while closer to HCC diagnosis (*p* = 0.024). The AUROC was 0.91 for predicting HCC within 1 year by including the predictors of age, sex, alanine aminotransferase, alpha-fetoprotein (AFP) and WFA^+^-M2BP.

**Conclusions:**

Serum WFA^+^-M2BP level may elevate before HCC onset and is a short-term predictor of HCC among patients infected with HCV.

## Introduction

Hepatitis C virus (HCV) infection is a considerable public health burden worldwide. Approximately 170–200 million persons currently have chronic HCV infection, and 3–4 million persons are newly infected annually^1^. Approximately 20% of patients with chronic HCV infection are at risk of liver cirrhosis within 25 years; cirrhosis in 25% of at-risk patients may subsequently progress to hepatocellular carcinoma (HCC). Annually, HCV accounts for at least 360,000 deaths because of liver cirrhosis or HCC^2^.

Patients at a high risk of HCC are recommended for clinical surveillance including ultrasonographic examination or the combined use of serum alpha-fetoprotein (AFP)^3–5^. However, the sensitivity and specificity of AFP (cut-off value, 20 ng/mL) in detecting HCC are approximately 0.6 and 0.9, respectively^6,7^, suggesting low AFP levels in some patients with HCC. Contrastingly, ultrasonography can reveal marked variations in sensitivity and specificity, depending on the experience of the operator^8^. Therefore, determination of useful biomarkers that may supplement routine clinical examinations is warranted.

Mac-2-binding protein (M2BP) is a cell adhesion protein of the extracellular matrix, which can self-assemble into ring-like structures and bind to certain integrins, collagens and fibronectins^9^. Recently, *Wisteria floribunda* agglutinin-positive human M2BP (WFA^+^-M2BP) was reported as a non-invasive glycomarker of liver fibrosis, and serum WFA^+^-M2BP levels were found to be significantly elevated at various stages of liver fibrosis^10^. In addition, elevated serum WFA^+^-M2BP levels were shown to be more a accurate predictor of HCC than was AFP^11^.

The Risk Evaluation of Viral Load Elevation and Associated Liver Disease/Cancer-HCV (REVEAL-HCV) cohort followed patients regularly through serial sampling. In the present study, we evaluated the short-term and long-term associations of serum WFA^+^-M2BP levels on the risk of new-onset HCC. According to our review of relevant literature, this is the first community-based longitudinal study to estimate the associations between serum WFA^+^-M2BP levels and HCC risk, with serial measurements of WFA^+^-M2BP levels.

## Methods

### Study participants and data collection

**Fig. 1:**
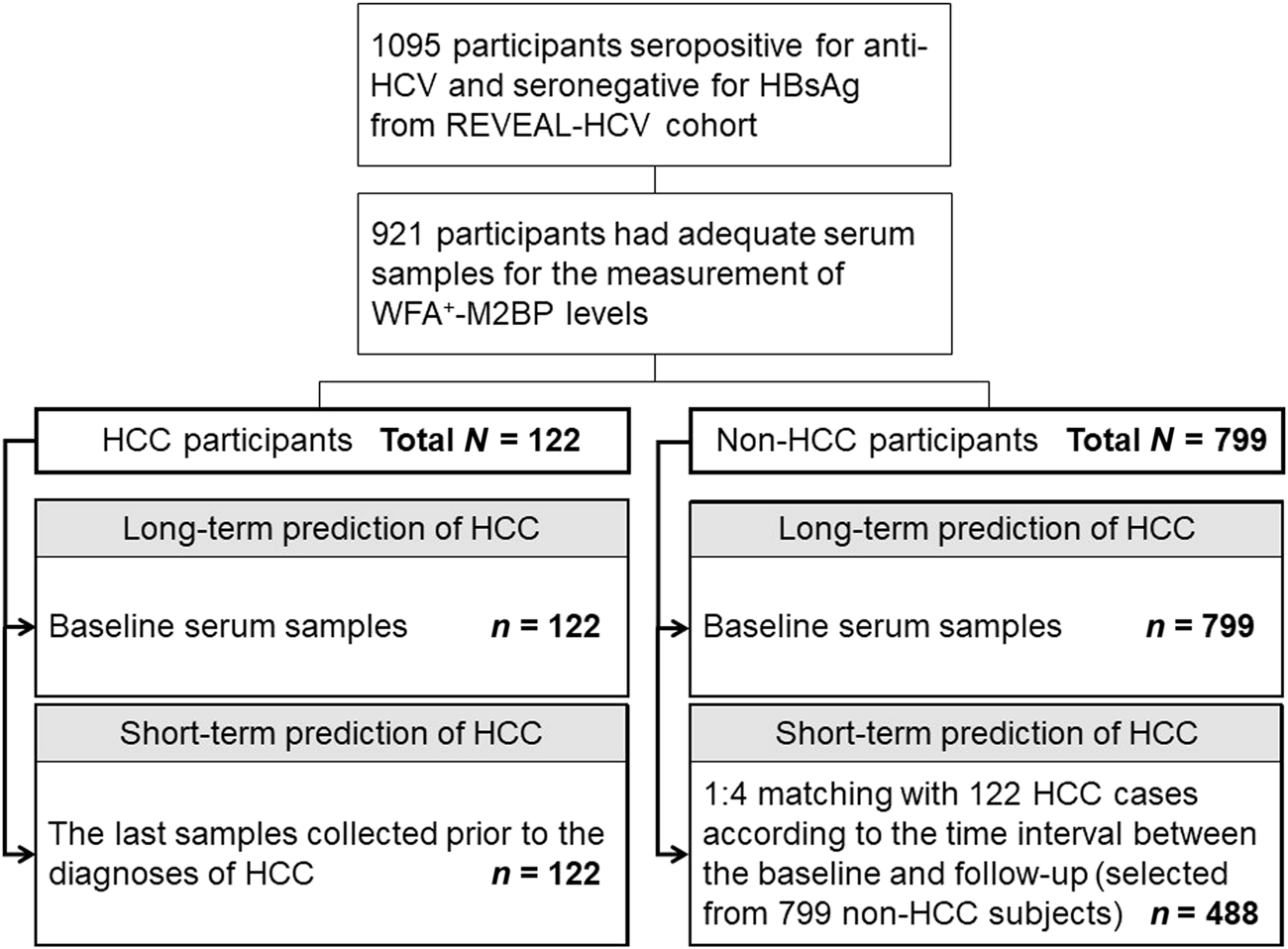
Flowchart of the study participants. Anti-HCV anti-hepatitis C virus antibody, HBsAg hepatitis B surface antigen, REVEAL-HCV The Risk Evaluation of Viral Load Elevation and Associated Liver Disease/Cancer-HCV, WFA^+^-M2BP *Wisteria floribunda* agglutinin-positive human Mac-2-binding protein, HCC hepatocellular carcinoma

Studies have reported the enrollment of participants in the REVEAL-HCV cohort^12,13^. Briefly, it is a community-based cohort of 1095 participants seropositive for antibodies against HCV (anti-HCV) and seronegative for hepatitis B surface antigen (HBsAg). The participants were enrolled from seven townships in Taiwan during 1991–1992. All participants provided written informed consent and were personally interviewed by trained public-health nurses who used a structured questionnaire. A 10-mL blood sample was collected from each participant upon enrollment. During 1991–2005, the participants were invited for health examinations and serological tests every 6–12 months. Serum levels of alanine aminotransferase (ALT), aspartate aminotransferase (AST) and AFP were regularly and serially measured. The fractionated serum samples were stored at −70 °C until assayed. In addition to the tests of seromarkers, the participants were examined by high-resolution abdominal ultrasonography by a certified gastroenterologist and interpreted according to a standardized protocol set by a specialist panel. Liver cirrhosis was determined based on a quantitative scoring system, which was derived from the appearance of the liver surface (normal, irregular, undulated), liver parenchymal texture (normal, heterogeneous, coarse), intrahepatic blood vessel size (normal, obscure, narrowing) and splenic size (normal, enlarged). The computerized data linkage with the National Health Insurance Database was performed to complete the ascertainment^14^. This study was approved by the institutional review board of the College of Public Health, National Taiwan University, Taipei. Among the 1095 participants, adequate serum samples were obtained from 921 participants for the baseline measurement of WFA^+^-M2BP levels and were included in the analysis. To evaluate the short-term associations between WFA^+^-M2BP levels and HCC, we retrieved the last follow-up samples in addition to the baseline serum samples. We utilized the repeated samples of the study subjects and conducted a nested case–control study. For HCC cases, we retrieved the last serum samples collected before the diagnosis of HCC. The controls were frequency-matched at a ratio of 1:4 according to the time interval between the baseline and last follow-up. Figure 1 shows the flowchart of the study subjects.

### Ascertainment of HCC

All the participants were free of HCC at study entry. New-onset HCC was ascertained through regular health examinations and computerized data linkage with the National Cancer Registration profiles from January 1, 1991 to December 31, 2013. To ensure complete ascertainment, computerized linkage with the National Death Certification profiles was used to identify deaths resulting from HCC. Participants with suspected HCC who were identified in regular health examinations were referred for further clinical examination, namely a liver biopsy, angiogram, or computed tomography. The HCC cases were confirmed through (1) histopathological examination; (2) two imaging techniques (abdominal ultrasonography, angiogram, or computed tomography) or (3) one imaging technique plus a serum AFP level of ≥ 400 ng/mL^15^.

### Laboratory examinations

Serological testing was performed as follows: ALT and AST were tested using an autoanalyser (Toshiba TBA-200FR, Japan), with commercial reagents (Denka Seiken, Tokyo, Japan). AFP was measured by radioimmunoassay with BRAHMS AFP KRYPTOR (Brahms France, Sartrouville, France). HBsAg was determined by radioimmunoassay with commercial kits (Abbott Laboratories, North Chicago, IL, USA) and anti-HCV was determined by enzyme immunoassay with commercial kits (Abbott Laboratories). Samples seropositive for anti-HCV were further measured for HCV RNA through polymerase chain reaction by performing the COBAS TaqMan HCV test, v2.0 (Roche Diagnostics, Indianapolis, NJ, USA). WFA^+^-M2BP levels were determined by sandwich immunoassay through an automatic immunoanalyser (HISCL-2000i, Sysmex Co., Tokyo, Japan), and the cut-off index (COI) of each sample was automatically calculated and provided by the instrument. Studies have reported the definition and standardization of the COI^10,11,16^. Briefly, the measured values of WFA^+^-M2BP conjugated to WFA were indexed by the following equation:

$${{\rm COI}} = \left( {\left[ {{{\rm WFA}}^ + - {{\rm M2BP}}} \right]_{{{\rm sample}}} - \left[ {{{\rm WFA}}^ + - {{\rm M2BP}}} \right]_{{{\rm NC}}}} \right)/\\ \left( {\left[ {{{\rm WFA}}^ + - {{\rm M2BP}}} \right]_{{{\rm PC}}}} \right. - \left( {\left[ {{{\rm WFA}}^ + - {{\rm M2BP}}} \right]_{{{\rm NC}}}} \right)$$,

where sample means the tested serum sample; PC means positive control and NC means negative control. The positive control was recombinant WFA^+^-M2BP from human embryonic kidney cells, which supplied as a calibration solution preliminarily standardized to yield a COI value of 1.0^16^.

### Statistical analysis

We evaluated both long-term and short-term associations of serum WFA^+^-M2BP levels with HCC risk. The person-years of follow-up were calculated for each participant from the entry date to either HCC diagnosis, death, or December 31, 2013 (whichever occurred first). The incidence rate of HCC is expressed per 100,000 person-years of follow-up. Cox proportional hazards models were applied to estimate the hazard ratios (HRs) with 95% confidence intervals (CIs) of various levels of WFA^+^-M2BP and the risk of HCC. In addition, the dose–response relationships between WFA^+^-M2BP levels and HCC were evaluated through trend tests. To evaluate the effects of increasing changes in WFA^+^-M2BP levels on HCC risk, serum WFA^+^-M2BP levels at baseline and the last follow-up were compared. The subtraction of serum WFA^+^-M2BP levels at two time points was divided by the number of years between the two time points to determine the extent of changes in WFA^+^-M2BP levels. A change of ≥ 0.3 COI was considered an increasing change^17^.

To determine the efficacy of WFA^+^-M2BP levels for predicting HCC at various time intervals before HCC diagnosis, non-parametric Mann–Kendall tests were conducted to examine the trends of the areas under receiver operating characteristics curves (AUROCs) of WFA^+^-M2BP for predicting HCC^18^. Logistic regression was used to estimate the odds ratios (ORs) with 95% CIs to evaluate the short-term associations between serum WFA^+^-M2BP levels and HCC risk. Stratification analysis was performed according to the time intervals between the HCC diagnosis date and last measurements of WFA^+^-M2BP levels. The time intervals before HCC diagnosis were stratified by < 1, 1–3, 3–6 and > 6 years to evaluate the magnitude of associations between serum WFA^+^-M2B levels and HCC risk at various time points. The cut-off WFA^+^-M2BP levels were determined by the highest Youden index (sensitivity + specificity − 1) among WFA^+^-M2BP levels of 1.0, 1.5 and 2.0. Multiple logistic regression models were used to estimate the adjusted ORs after adjustment for age, sex, serum ALT levels and AST/ALT ratio at the time points of WFA^+^-M2BP testing.

The efficacy of serum WFA^+^-M2BP and AFP levels for predicting HCC was compared for the various time points. The AUROCs at various time points were estimated using three models. Model 1 included age, sex, serum levels of ALT, AST/ALT ratio and WFA^+^-M2BP; Model 2 included age, sex, serum levels of ALT, AST/ALT ratio and AFP; Model 3 included all of the considered risk factors in Model 1 and Model 2. Venkatraman’s tests were used to examine and compare the differences in the AUROCs for the three prediction models for HCC with or without WFA^+^-M2BP levels^19^. The associations between AFP and WFA^+^-M2BP were estimated by Pearson correlation coefficients. All tests were two-tailed tests, and results with *p* < 0.05 were considered statistically significant. All analyses were performed using SAS statistical software (Version 9.3; SAS Institute Inc., Cary, NC, USA).

## Results

### Long-term associations between WFA^+^-M2BP levels and HCC

Table 1 summarizes the baseline characteristics of the 921 participants. The median follow-up of the participants was 21.7 years. A total of 122 new-onset HCC cases were identified throughout 17,561 person-years, giving an HCC incidence of 694.7 per 100,000 person-years. Older age; male sex; liver cirrhosis and elevated serum levels of ALT, AST, AFP and HCV RNA were predictors of HCV-related HCC. Moreover, elevated serum WFA^+^-M2BP levels at study entry were found to be a long-term predictor of HCC (*p* < 0.001 for the trend). The adjusted HRs (95% CIs) were 1.63 (0.89–3.00), 3.03 (1.62–5.70), 3.77 (1.79–7.95) and 4.10 (1.81–9.27) for COIs of 0.5–1.0, 1.0–1.5, 1.5–2.0 and ≥ 2.0, respectively (WFA^+^-M2BP < 0.5 COI was considered as the reference group).

**Table 1:** Incidence rates and hazard ratios of HCC by characteristics or serum markers at baseline

Baseline demographic or characteristic^+^	Number of participants (*n* = 921)	Person-years of follow-up (total = 17,561)	Number of HCC cases (*n* = 122)	Incidence rate per 100,000 person-years	Crude hazard ratio (95% confidence interval)	*P*-value	Adjusted hazard ratio* (95% confidence interval)	*P*-value
Age								
30–39	167	3507.54	6	171.06	1.00		1.00	
40–49	203	4163.47	12	288.22	1.72 (0.65–4.59)	0.2771	1.54 (0.56–4.25)	0.4032
50–59	370	6738.41	72	1068.50	6.89 (2.99–15.84)	<0.0001	5.07 (2.18–11.78)	0.0002
60+	181	3151.21	32	1015.48	6.97 (2.91–16.69)	<0.0001	5.62 (2.32–13.60)	0.0001
Sex								
Female	544	10745.00	58	539.79	1.00		1.00	
Male	377	6815.63	64	939.02	1.83 (1.29–2.62)	0.0008	1.52 (1.02–2.26)	0.0402
Liver cirrhosis								
No	906	17348.32	117	674.42	1.00		1.00	
Yes	11	134.98	5	3704.25	7.18 (2.92–17.64)	<0.0001	3.56 (1.17–10.85)	0.0257
ALT								
<15 U/L	389	7712.01	30	389.00	1.00		1.00	
15–45 U/L	379	7184.15	55	765.57	2.01 (1.29–3.14)	0.0021	1.72 (1.07–2.78)	0.0265
≥45 U/L	146	2528.99	37	1463.03	4.03 (2.49–6.53)	<0.0001	2.69 (1.49–4.87)	0.0011
AST								
<15 U/L	239	4845.11	7	144.48	1.00		1.00	
15–45 U/L	534	10312.41	78	756.37	5.33 (2.46–11.55)	<0.0001	3.89 (1.78–8.51)	0.0007
≥45 U/L	141	2267.64	37	1631.65	12.71 (5.66–28.53)	<0.0001	8.31 (3.4–20.30)	<0.0001
AFP								
<5 ng/mL	690	13497.02	71	526.04	1.00		1.00	
5–10 ng/mL	164	2988.40	34	1137.73	2.28 (1.51–3.43)	<0.0001	1.87 (1.20–2.90)	0.0055
≥10 ng/mL	65	1049.69	16	1524.26	3.29 (1.91–5.66)	<0.0001	2.28 (1.22–4.26)	0.0099
Serum HCV RNA viral load								
<25 (IU/mL)	277	5689.00	8	140.62	1.00		1.00	
25–23,000 (IU/mL)	284	5154.79	47	911.77	6.96 (3.29–14.73)	<0.0001	5.63 (2.62–12.09)	<0.0001
≥23,000 (IU/mL)	281	5169.95	57	1102.53	8.43 (4.02–17.67)	<0.0001	6.81 (3.23–14.36)	<0.0001
WFA^+^-M2BP								
<0.5	257	5285.50	16	302.71	1.00		1.00	
0.5– < 1.0	380	7396.73	40	540.78	1.86 (1.04–3.32)	0.0364	1.63 (0.89–3.00)	0.1132
1.0– < 1.5	166	3011.81	35	1162.09	4.21 (2.33–7.61)	<0.0001	3.03 (1.62–5.70)	0.0005
1.5– < 2.0	68	1147.16	15	1307.58	4.97 (2.46–10.06)	<0.0001	3.77 (1.79–7.95)	0.0005
≥2.0	50	719.43	16	2223.98	9.38 (4.68–18.78)	<0.0001	4.10 (1.81–9.27)	0.0007

*Adjustment of all risk factors, i.e. age, sex, liver cirrhosis status, levels of ALT, AST, AFP, serum HCV RNA viral load and WFA^+^-M2BP

*HCC* hepatocellular carcinoma, *WFA*^*+*^*-M2BP* *Wisteria floribunda* agglutinin-positive human Mac-2-binding protein, *ALT* alanine aminotransferase, *AST* aspartate aminotransferase, *AFP* alpha-fetoprotein, *HCV* hepatitis C virus

### Changes in serum WFA^+^-M2BP levels at study entry and the last follow-up

**Fig. 2:**
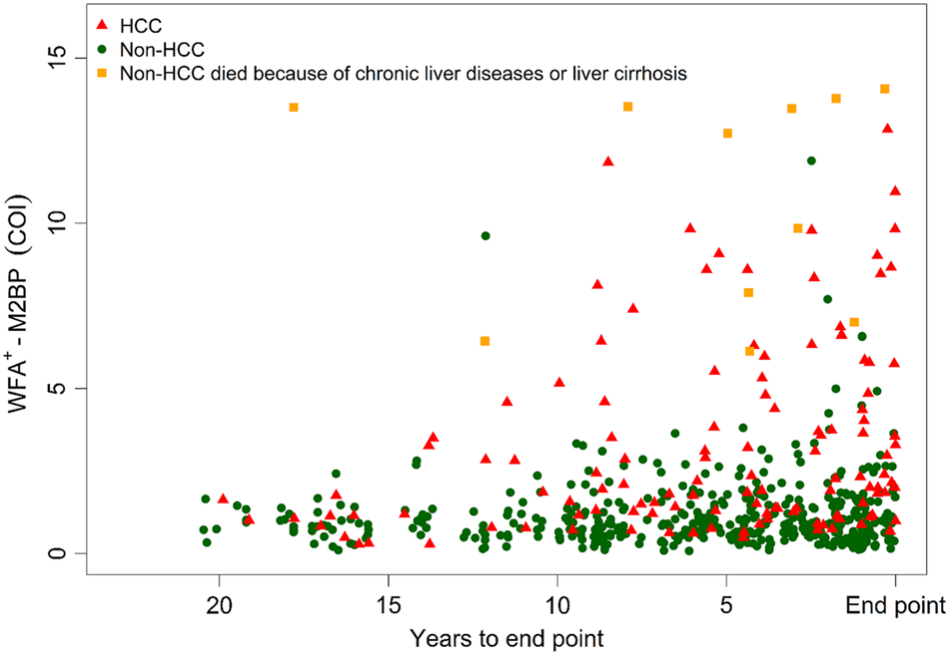
Distribution of serum WFA^+^-M2BP levels stratified by the time before HCC diagnosis

Most HCC cases exhibited an increasing trend of WFA^+^-M2BP levels between baseline and the last follow-up. Contrastingly, most non-HCC participants showed low WFA^+^-M2BP levels, and the changes between the two time points were limited. Some non-HCC participants showed significantly increasing changes in WFA^+^-M2BP levels. However, they died because of chronic liver disease or cirrhosis during follow-up. Figure 2 shows the WFA^+^-M2BP levels of HCC cases stratified by the time points of the WFA^+^-M2BP tests before HCC diagnosis. Most HCC cases had elevated WFA^+^-M2BP levels 10 years before diagnosis; their serum WFA^+^-M2BP levels were even higher when their last tests were conducted closer to the diagnosis date. In the figure, WFA^+^-M2BP levels of non-HCC participants are marked with green dots. Most non-HCC participants showed low WFA^+^-M2BP levels. Non-HCC participants with high WFA^+^-M2BP levels were those who died because of chronic liver disease or cirrhosis.

### Increasing WFA^+^-M2BP trajectory of serial tests and HCC risk

We compared the WFA^+^-M2BP levels at baseline and the last follow-up to determine the changes in the trajectories of serum WFA^+^M2BP levels and HCC risk. Most HCC cases exhibited an increasing trend of WFA^+^-M2BP levels between the two time points. We further compared the participants with increasing WFA^+^-M2BP levels equal to or higher than 0.3 COI and those with increasing WFA^+^-M2BP levels lower than 0.3 COI from baseline to the last follow-up. Furthermore, 24.8% of HCC cases showed increasing WFA^+^-M2BP levels equal to or higher than 0.3 COI compared with 7.9% of non-HCC participants. After adjustment for age; sex; status of liver cirrhosis and serum levels of ALT, AST, AFP and HCV RNA, the HR for HCC was 4.36 (95% CI: 2.61–7.29; *p* < 0.001) for the participants with increasing WFA^+^-M2BP levels equal to or higher than 0.3 COI compared with that for the participants with increasing WFA^+^-M2BP levels less than 0.3 COI.

### Efficacy of M2BP for predicting HCC at various time intervals

**Fig. 3:**
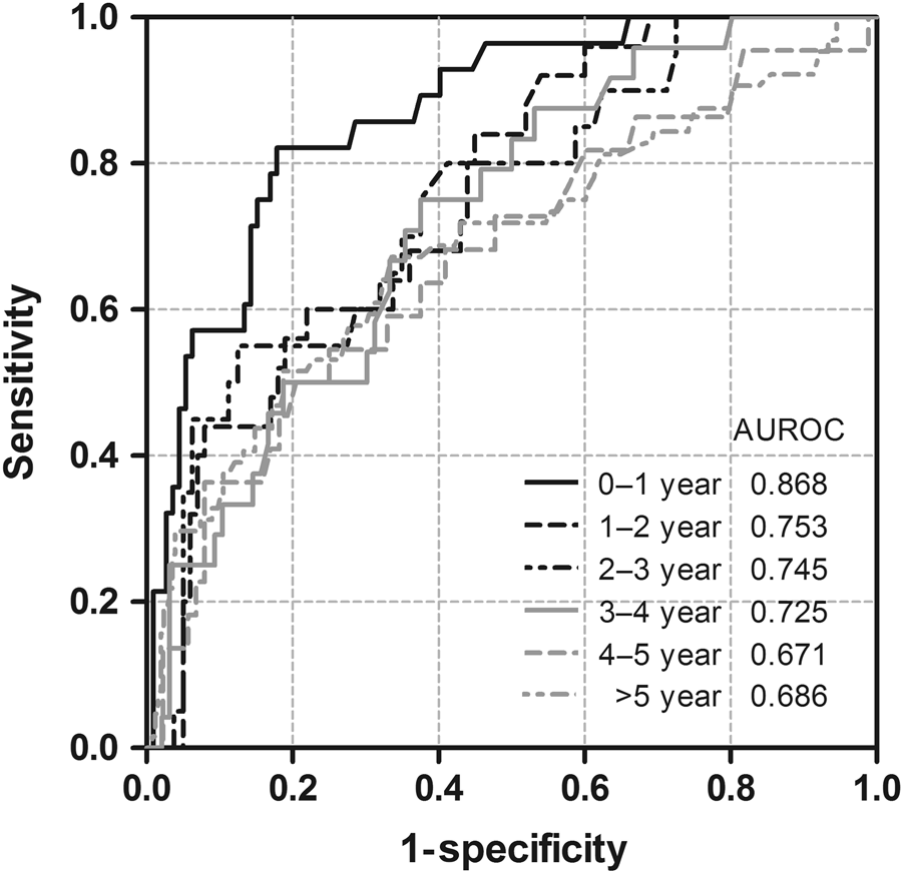
ROC curves and AUROCs of serum WFA^+^-M2BP levels for predicting HCC at various time points

We estimated AUROCs to determine the efficacy of WFA^+^-M2BP levels for predicting HCC. The overall AUROC of WFA^+^-M2BP was 0.76 (95% CI: 0.72–0.81). Figure 3 shows that the HCC predictability of WFA^+^-M2BP gradually decreased with longer intervals between WFA^+^-M2BP tests and the HCC diagnosis date (*p* = 0.024). For the prediction of HCC within 1 year, the AUROC of WFA^+^-M2BP was 0.87 (95% CI: 0.80–0.94). Considering 1.5 COI as a cut-off WFA^+^-M2BP level, the sensitivity and specificity were 0.86 and 0.71, respectively, when WFA^+^-M2BP levels were last measured within 1 year before HCC diagnosis. On the other hand, the AUROC of AFP for predicting HCC within 1 year was 0.82 (95% CI: 0.72–0.92). By using 20 ng/mL as the cut-off value for AFP, the sensitivity was 0.36 and the specificity was 0.98; by setting the cut-off value as 5 ng/mL, both sensitivity and specificity were 0.75 for AFP for the prediction of HCC. This finding suggested that the short-term efficacy of WFA^+^-M2BP levels for predicting HCC was satisfactory.

### Magnitude of associations between M2BP and HCC by various time intervals

Table 2 shows the crude and adjusted ORs of WFA^+^-M2BP levels for HCC risk stratified by time intervals of < 1, 1–3, 3–6 and > 6 years between the date of HCC diagnosis and that of the last measurements of WFA^+^-M2BP levels. Participants with WFA^+^-M2BP levels higher than 1.5 COI were compared with those with WFA^+^-M2BP levels equal to or lower than 1.5 COI. Higher WFA^+^-M2BP levels were associated with an elevated HCC risk in each group after adjustment for age, sex, serum levels of ALT and AST/ALT ratio. The adjusted ORs of WFA^+^-M2BP levels for HCC risk were the highest when the WFA^+^-M2BP test was performed closer to HCC diagnosis (within 1 year). The adjusted OR (95% CI) associated with HCC within 1 year of WFA^+^-M2BP levels was 7.95 (2.25–28.04). Considering 1.5 COI as the cut-off WFA^+^-M2BP level, the sensitivity and specificity were, respectively, 0.86 and 0.71 when the WFA^+^-M2BP levels were last measured within 1 year before HCC diagnosis.

**Table 2:** Associations of serum WFA^+^-M2BP levels with HCC stratified by the time before HCC diagnosis

Time interval before diagnosis of HCC	HCC risk by WFA^+^-M2BP level of 1.5 COI			
	Crude OR (95% CI)	*P*-value	Adjusted OR* (95% CI)	*P*-value
0–1 year	15.00 (4.82–46.67)	<0.0001	7.95 (2.25–28.04)	0.001
1–3 years	4.14 (1.98–8.67)	0.0002	2.55 (1.08–5.99)	0.032
3–6 years	3.87 (2.05–7.32)	<0.0001	1.96 (0.96–4.01)	0.065
>6 years	4.42 (2.31–8.49)	<0.0001	2.78 (1.30–5.95)	0.008

*Adjustment of age, sex, ALT and AST/ALT ratio

*WFA*^*+*^*-M2BP* Wisteria floribunda agglutinin-positive human Mac-2-binding protein, *HCC* hepatocellular carcinoma, *ALT* alanine aminotransferase, *AST* aspartate aminotransferase

### Serum levels of M2BP and AFP and the prediction of HCC

Table 3 shows the AUROCs for predicting HCC, as stratified by various time intervals with three different models. Model 1 included age, sex, serum levels of ALT, AST/ALT ratio and WFA^+^-M2BP; Model 2 replaced WFA^+^-M2BP with AFP and Model 3 included all risk factors, as well as WFA^+^-M2BP and AFP. All the three models showed that the AUROCs gradually decreased with longer time intervals before HCC diagnosis. The AUROCs of Model 1 and Model 2 were similar at each time point, suggesting that the efficacy of WFA^+^-M2BP for predicting HCC was comparable to that of AFP. However, the AUROCs were 0.86 (95% CI: 0.78–0.95) for Model 2 and 0.91 (95% CI: 0.85–0.97) for Model 3 for the 0–1-year prediction of HCC, indicating that including WFA^+^-M2BP increased the short-term predictability of HCC (*p* = 0.033). In addition, the AUROC of the complete model was 0.95 (0.90–1.00) for predicting HCC within 1 year among the patients without cirrhosis, which implicated that the increasing serum WFA^+^-M2BP levels was associated with hepatocarcinogenesis. In contrast, our results did not reveal any extra predictability of HCC among patients with cirrhosis, as indicated by a comparison between the models with and without serum WFA^+^-M2BP. Moreover, the association between AFP and WFA^+^-M2BP were low (*r* = 0.09 for baseline WFA^+^-M2BP and AFP; *r* = 0.02 for follow-up WFA^+^-M2BP and AFP), suggesting that WFA^+^-M2BP levels could provide additional information for liver disease and could be used as a useful adjunct marker.

**Table 3:** AUROCs of the prediction models of age, sex, ALT and AST/ALT ratio with WFA^+^-M2BP or AFP or both

Time interval before diagnosis of HCC	Model 1	Model 2	Model 3	*P*-value^+^
0–1 year	0.885	0.863	0.913	0.033
1–3 years	0.826	0.836	0.847	0.256
3–6 years	0.808	0.820	0.835	0.280
>6 years	0.794	0.786	0.809	0.423

Model 1: including age, sex, ALT, AST/ALT ratio and WFA^+^-M2BP; Model 2: including age, sex, ALT and AST/ALT ratio, AFP; Model 3: including age, sex, ALT, AST/ALT ratio, WFA^+^-M2BP and AFP as predictors. + : Comparison of predictability by AUROC between Model 2 and Model 3

*ALT* alanine aminotransferase, *AST* aspartate aminotransferase, *WFA*^*+*^*-M2BP* Wisteria floribunda agglutinin-positive human Mac-2-binding protein, *AFP* alpha-fetoprotein, *HCC* hepatocellular carcinoma

## Discussion

In this community-based longitudinal study, we found that serum WFA^+^-M2BP levels were both long- and short-term predictors of HCC in the patients infected with HCV. Serum WFA^+^-M2BP levels may increase before HCC development. In addition, the prediction efficacy of WFA^+^-M2BP levels was markedly higher within 1 year before HCC diagnosis compared with that at later time points.

The clinical utility of WFA^+^-M2BP levels has been evaluated in patients with various diagnoses^10,11,17,20–25^. Serum WFA^+^-M2BP levels have been suggested as a biomarker for HCV-related liver fibrosis^10,20^ and HCC^11,17^. Patients with elevated serum levels of WFA^+^-M2BP were shown to have higher hepatic fibrosis scores^11^. Among patients with chronic HCV infection who received direct-acting antiviral treatment, high serum levels of WFA^+^-M2BP reduced the sustained virological response rate^22^. In addition to patients infected with HCV, M2BP levels were an independent predictor of HCC among patients with chronic hepatitis B^24^. Among patients with HCC who had undergone liver surgery, elevated serum levels of WFA^+^-M2BP at the time of surgery were found to increase the tumour recurrence risk and result in poor overall survival after surgery^21^. Overall, this evidence indicates the high potential of WFA^+^-M2BP levels for the risk stratification of patients with liver disease.

Most related studies have reported that the effects of WFA^+^-M2BP levels on the risk of liver fibrosis or HCC were based on clinical patients^10,11,17,20,26^. One of those studies evaluated the changes in WFA^+^-M2BP over time^17^. They compared the WFA^+^-M2BP levels at study entry and the last follow-up. Furthermore, they measured the magnitude of changes in WFA^+^-M2BP levels. Consistent with their findings, our data reveal that patients with increasing levels of WFA^+^-M2BP equal to or higher than 0.3 COI had a 4.36-fold risk of HCC. Compared with the serum WFA^+^-M2BP levels of patients in previous studies^11,17^, those of our patients were relatively low. Only 1% of our patients had serum WFA^+^-M2BP levels equal to or higher than 4.0 COI at study entry. However, 17–20% of the patients showed M2BP levels higher than 4.0 COI in previous studies^11,17^. The main reason for this difference is that our study enrolled community-residing patients. Most of them were asymptomatic and relatively healthier than clinical patients recruited from hospitals.

In addition to measuring the baseline serum WFA^+^-M2BP levels for one-shot tests, we repeatedly measured serum samples; thus, the changes in the trajectory of serum WFA^+^-M2BP levels and their associations with the risk of HCC were determined. However, how this marker fluctuates over time during the infection course remains unknown. The associations of serial measurements with serum levels of WFA^+^-M2BP, ALT, AST and AFP may facilitate clarifying the role of WFA^+^-M2BP during liver disease progression. In the long run, HCC may subsequently develop in some patients who continue to experience treatment-induced RNA clearance^27^. The changing patterns of serum WFA^+^-M2BP levels in these patients may provide information on the utility of this marker for HCC surveillance.

REVEAL-HCV cohort is recognized as a natural history cohort^13,28^. Most of the participants did not have the experience of antiviral treatment due to its high cost and adverse effects previously. Until October 2003, only patients with ALT levels higher than 82 U/L and moderate fibrosis proven by liver biopsy could be reimbursed for interferon-based treatment by the National Health Insurance. Among 921 participants with seropositive anti-HCV in this study, 111 participants died before the end of 2003. There were 39 patients with elevated serum ALT levels ( > 82 U/L) in the remaining 810 participants. However, it was not practical to perform liver biopsy examination for these asymptomatic subjects in the community. Although the newly direct antiviral agents started to be reimbursed since January 2017, only patients with advanced fibrosis stage ( ≧ F3) could receive the treatments. Moreover, there are still huge gaps of self-awareness, referral and linking to care. In Taiwan, 36% of anti-HCV seropositives had disease awareness. Among those with awareness, 40% had accessibility for antivirals, and the acceptance rate of antivirals was around 70%^29^. Therefore, treatment rate was around 10%, suggesting that there were less than four subjects who might have treatment experiences among the 921 participants. Thus, we consider that this cohort reflected the natural fluctuations of serum levels of WFA^+^-M2BP and liver diseases. However, as HCV antivirals will become more and more accessible in Taiwan, we may perform computerized data linkage with the National Health Insurance Database to evaluate the proportions of subjects with antiviral treatment in the future.

As we know, it is relevant to evaluate the applicability of WFA^+^-M2BP on various degrees of progression of HCC. However, the newly onset of HCC in this study was ascertained by regular health examinations and computerized data linkage with the National Cancer Registration profiles and National Death Certification profiles. The information of tumour size or tumour numbers were not obtainable, which limited the possibility to examine the associations of WFA^+^-M2BP on the severity of HCC. We invited the participants to return for health check every 6–12 months in the community regularly by phone calls and mailing letters. These asymptomatic individuals who had willingness to return were those who were more motivated.

This community-based study, by enrolling relatively health subjects with HCV infection, lacked liver biopsy and serum levels of platelet counts. The measurements of platelet counts could not be examined by using the stored samples. Therefore, the information of liver fibrosis was not available. We used ALT/AST ratio as a surrogate of fibrotic marker in the analyses. There were more than 80% of newly developed HCC among anti-HCV seropositives that may already have liver cirrhosis detected by ultrasonography or an increased ratio between serum levels of AST to ALT. The occurrence of liver cirrhosis is an intermediate stage that occurs in the natural history of HCC among patients with HCV infection. Therefore, it is not easy to clearly differentiate whether the levels of WFA^+^-M2BP are associated with fibrosis, liver cirrhosis and then the subsequent risk of HCC. However, based on the previous study, the predictability of HCC by WFA^+^M2BP was still high, even stratified by varied levels of liver fibrosis^11^. In our study, we found that the levels of WFA^+^-M2BP played as a long-term as well as a short-term marker for HCC, suggesting that the applicability of this surrogate marker may be increased when applied to asymptomatic subjects with HCV infection living in the community.

In conclusion, our study results suggest that the serum level of WFA^+^-M2BP is both a short-term and long-term predictor of HCV-related HCC. Combining the satisfactory sensitivity and specificity of WFA^+^-M2BP and AFP, respectively, suggested a more accurate prediction of the occurrence of HCC. Intensive care must therefore be provided to patients with elevated serum WFA^+^-M2BP levels.

### Study Highlights

#### What is current knowledge

- Patients at high risk of HCC are recommended for clinical surveillance.
- Serum levels of WFA^+^-M2BP were found to increase significantly with degrees of liver fibrosis among clinical patients.

#### What is new here

- Serum WFA^+^-M2BP levels had a significant increasing trend before the occurrence of HCC.
- The predictability of HCC by WFA^+^-M2BP increased with shorter time intervals before HCC diagnosis.
- WFA^+^-M2BP is a long-term predictor as well as a short-term predictor of HCC among HCV-infected patients.

## References

[1] Mohd Hanafiah K (2013). Global epidemiology of hepatitis C virus infection: new estimates of age-specific antibody to HCV seroprevalence. Hepatology.

[2] Perz JF (2006). The contributions of hepatitis B virus and hepatitis C virus infections to cirrhosis and primary liver cancer worldwide. J. Hepatol..

[3] Bruix J (2006). New aspects of diagnosis and therapy of hepatocellular carcinoma. Oncogene.

[4] Maruyama H, Yoshikawa M, Yokosuka O (2008). Current role of ultrasound for the management of hepatocellular carcinoma. World J. Gastroenterol..

[5] Bolondi L (2001). Surveillance programme of cirrhotic patients for early diagnosis and treatment of hepatocellular carcinoma: a cost effectiveness analysis. Gut.

[6] Trevisani F (2001). Serum alpha-fetoprotein for diagnosis of hepatocellular carcinoma in patients with chronic liver disease: influence of HBsAg and anti-HCV status. J. Hepatol..

[7] Sherman M, Peltekian KM, Lee C (1995). Screening for hepatocellular carcinoma in chronic carriers of hepatitis B virus: incidence and prevalence of hepatocellular carcinoma in a North American urban population. Hepatology.

[8] Daniele B (2004). Alpha-fetoprotein and ultrasonography screening for hepatocellular carcinoma. Gastroenterology.

[9] Sasaki T (1998). Mac-2 binding protein is a cell-adhesive protein of the extracellular matrix which self-assembles into ring-like structures and binds beta1 integrins, collagens and fibronectin. EMBO J..

[10] Kuno A (2013). A serum “sweet-doughnut” protein facilitates fibrosis evaluation and therapy assessment in patients with viral hepatitis. Sci. Rep..

[11] Yamasaki K (2014). Elevated serum levels of Wisteria floribunda agglutinin-positive human Mac-2 binding protein predict the development of hepatocellular carcinoma in hepatitis C patients. Hepatology.

[12] Lee MH (2011). Community and personal risk factors for hepatitis C virus infection: a survey of 23,820 residents in Taiwan in 1991-2. Gut.

[13] Lee MH (2010). Hepatitis C virus seromarkers and subsequent risk of hepatocellular carcinoma: long-term predictors from a community-based cohort study. J. Clin. Oncol..

[14] Lee MH (2013). Prediction models of long-term cirrhosis and hepatocellular carcinoma risk in chronic hepatitis B patients: risk scores integrating host and virus profiles. Hepatology.

[15] Bruix Jordi, Sherman Morris, Llovet Josep M, Beaugrand Michel, Lencioni Riccardo, Burroughs Andrew K, Christensen Erik, Pagliaro Luigi, Colombo Massimo, Rodés Juan (2001). Clinical Management of Hepatocellular Carcinoma. Conclusions of the Barcelona-2000 EASL Conference. Journal of Hepatology.

[16] Kuno A (2013). Reconstruction of a robust glycodiagnostic agent supported by multiple lectin-assisted glycan profiling. Proteom. Clin. Appl..

[17] Tamaki N (2015). Wisteria floribunda agglutinin positive human Mac-2-binding protein as a predictor of hepatocellular carcinoma development in chronic hepatitis C patients. Hepatol. Res..

[18] Hirsch RM, Slack JR, Smith RA (1982). Techniques for trend assessment for monthly water quality data. Water Resour. Res..

[19] Venkatraman ES, Colin BB (1996). A distribution-free procedure for comparing receiver operating characteristic curves from a paired experiment. Biometrika.

[20] Toshima T (2015). A novel serum marker, glycosylated Wisteria floribunda agglutinin-positive Mac-2 binding protein (WFA( + )-M2BP), for assessing liver fibrosis. J. Gastroenterol..

[21] Fujiyoshi M (2015). Clinicopathological characteristics and diagnostic performance of Wisteria floribunda agglutinin positive Mac-2-binding protein as a preoperative serum marker of liver fibrosis in hepatocellular carcinoma. J. Gastroenterol..

[22] Ura K (2016). Serum WFA( + ) -M2BP is a non-invasive liver fibrosis marker that can predict the efficacy of direct-acting anti-viral-based triple therapy for chronic hepatitis C. Aliment. Pharmacol. Ther..

[23] Nagata Hiroko, Nakagawa Mina, Nishimura-Sakurai Yuki, Asano Yu, Tsunoda Tomoyuki, Miyoshi Masato, Kaneko Shun, Goto Fumio, Otani Satoshi, Kawai-Kitahata Fukiko, Murakawa Miyako, Nitta Sayuri, Itsui Yasuhiro, Azuma Seishin, Kakinuma Sei, Tojo Naoko, Tohda Shuji, Asahina Yasuhiro, Watanabe Mamoru (2016). Serial measurement of Wisteria floribunda agglutinin positive Mac-2-binding protein is useful for predicting liver fibrosis and the development of hepatocellular carcinoma in chronic hepatitis C patients treated with IFN-based and IFN-free therapy. Hepatology International.

[24] Ichikawa Yuki, Joshita Satoru, Umemura Takeji, Shobugawa Yugo, Usami Yoko, Shibata Soichiro, Yamazaki Tomoo, Fujimori Naoyuki, Komatsu Michiharu, Matsumoto Akihiro, Tanaka Eiji (2016). SerumWisteria floribundaagglutinin-positive human Mac-2 binding protein may predict liver fibrosis and progression to hepatocellular carcinoma in patients with chronic hepatitis B virus infection. Hepatology Research.

[25] Kono M (2016). Increased levels of serum Wisteria floribunda agglutinin-positive Mac-2 binding protein in idiopathic pulmonary fibrosis. Respir. Med..

[26] Iio E (2016). A novel glycobiomarker, Wisteria floribunda agglutinin macrophage colony-stimulating factor receptor, for predicting carcinogenesis of liver cirrhosis. Int. J. Cancer.

[27] Asahina Y (2013). lpha-fetoprotein levels after interferon therapy and risk of hepatocarcinogenesis in chronic hepatitis C. Hepatology.

[28] Lee MH (2012). Chronic hepatitis C virus infection increases mortality from hepatic and extrahepatic diseases: a community-based long-term prospective study. J. Infect. Dis..

[29] Yu ML (2015). Huge gap between clinical efficacy and community effectiveness in the treatment of chronic hepatitis C: a nationwide survey in Taiwan. Med. (Baltim.).

